# Quantifying motor adaptation in a sport-specific table tennis setting

**DOI:** 10.1038/s41598-023-50927-2

**Published:** 2024-01-05

**Authors:** Daniel Carius, Elisabeth Kaminski, Martina Clauß, Yannick Schewe, Lenja Ryk, Patrick Ragert

**Affiliations:** 1 Department of Movement Neuroscience, Faculty of Sport Science, Leipzig University, 04109 Leipzig, Germany; 2 Max Planck Institute for Human Cognitive and Brain Sciences, 04103 Leipzig, Germany

**Keywords:** Human behaviour, Learning and memory, Sensorimotor processing

## Abstract

Studies on motor adaptation aim to better understand the remarkable, largely implicit capacity of humans to adjust to changing environmental conditions. So far, this phenomenon has mainly been investigated in highly controlled laboratory setting, allowing only limited conclusions and consequences for everyday life scenarios. Natural movement tasks performed under externally valid conditions would provide important support on the transferability of recent laboratory findings. Therefore, one major goal of the current study was to create and assess a new table tennis paradigm mapping motor adaptation in a more natural and sport-specific setting. High-speed cinematographic measurements were used to determine target accuracy in a motor adaptation table tennis paradigm in 30 right-handed participants. In addition, we investigated if motor adaptation was affected by temporal order of perturbations (serial vs. random practice). In summary, we were able to confirm and reproduce typical motor adaptation effects in a sport-specific setting. We found, according to previous findings, an increase in target errors with perturbation onset that decreased during motor adaptation. Furthermore, we observed an increase in target errors with perturbation offset (after-effect) that decrease subsequently during washout phase. More importantly, this motor adaptation phenomenon did not differ when comparing serial vs. random perturbation conditions.

## Introduction

Over the past decades, motor adaptation has been subject of numerous behavioral and neurobehavioral studies, some of which have received considerable attention beyond movement neuroscience^1–5^. However, these neurobehavioral studies have exclusively been conducted in highly controlled laboratory settings, thus their methodological approach may interfere with the idea of ecological validity^6^.

In general, participants show motor adaptation when a perturbation, such as a varying force field^1,2,5,7–10^ or visuomotor rotation^3,4,11–15^ occurs during performance of a simple motor task. Perturbations are considered uncertainties of possible outcomes between an action and its corresponding feedback such as performing a grasp movement with one’s upper arm while hitting an object. These perturbations are intended to be comparable to the feeling of interacting with real-world objects that have various physical characteristics^16^. Target errors during task performance are caused by an abrupt introduction of a perturbation, but these errors progressively disappear over the course of trials when individuals modify their motor output to account for the respective perturbation. When perturbations disappear, transient after-effects occur. These after-effects, which again represent target errors, are considered evidence for adapted internal models^16^.

One objective of previous research was to elucidate factors influencing motor adaptation performance. Influencing factors investigated include age^13,15,17–24^, neurological diseases^20,24–30^, task-related factors such as force^31–33^, direction^1,5,7,9,32,34–36^ and interference^1,8,9,35,37^ or strategies such as feedback^4,34,38,39^, cueing^10,35,36^ and inhibition^12,40,41^. In summary, there is a large body of research that disclosed potential factors during motor adaptation to a certain perturbation. However, in more naturalistic environments, adaptations to multiple perturbations usually occur. Dual adaptation studies investigated interference in opposing tasks^1,37^. The extent to which interference can be reduced by sensory cues has also been investigated^35^. On the other hand, less research has been done on the temporal order of perturbations during motor adaptation^42^. In motor adaptation research, only blocked vs. random practice schedules have been investigated^42^. The influence of serial practice on motor adaptation has to our knowledge not yet been investigated thoroughly. According to studies on the contextual interference effect in motor learning, contextual interference is lower when practicing in serial order than in random order^43,44^. Therefore, it is tempting to speculate, that serial vs. random practice creates similar effects in a motor adaptation paradigm.

To the best of our knowledge motor adaptation studies were all conducted in laboratory settings. Best-known experimental paradigms observed force-field adaptations with robotic manipulators^1,2,5,7–10^ or visuomotor adaptations with computer-based devices that provide rotated visual feedback^3,4,11–15^. Robotic manipulators and computer-based devices have the advantage to investigate well controlled movements with a high level of standardization but mostly only allow observations of hand or arm movements. In addition, visuomotor adaptations were investigated using prism glasses^21–23,29,30^. Prism glasses also enable observation of adaptations during walking tasks^21,23,30^ but only under specific, secured laboratory conditions. Aside from these studies, very few studies have investigated adaptations in whole-body movements using virtual reality^45,46^, split-belts^24^ or stewart platforms^47^. However, these different paradigms all represent artificial motion tasks. Natural cognition is constrained in laboratory-based investigations by usage of artificial stimuli and simplified motor responses^6,48^. This reductionist methodology somewhat conflicts characteristics of real-world scenarios, such as sport-specific movements. The question of whether measurements and actions under laboratory conditions are comparable to behavior in real world remains unanswered. Methodological strategies employed thus far could jeopardize the concept of ecological validity^6^. Currently, it is recommended that future studies should increase ecological validity by moving from reductionist, artificial environments to complex, natural environments, including real-world movements, e.g. typical athletic movements^6,49^.

To develop a methodological basis for the investigation of motor adaptation under externally valid conditions, a suitable movement task is needed. Nevertheless, this movement task should be feasible under controlled laboratory settings and should provide access for neurophysiological methods to unravel neural correlates. In the present study, we aimed investigating motor adaptation in ecologically valid natural settings. For this reason, we developed a table tennis paradigm that is intended to provide best possible internal validity in an ecologically valid natural setting. In addition, we investigated if motor adaptation was affected by temporal order of perturbations (serial vs. random practice).

Table tennis has already been used as a suitable movement task in a large number of behavioral^50–52^ and neurophysiological studies^53–60^. Table tennis stroke movements are sport-specific, whole-body movement tasks with high demands on movement precision and timing. Furthermore, playing table tennis requires constant adjustments of movement patterns, since returning ball is always characterized by different speeds, directions and spins in constantly changing gaming situation.

Therefore, the main aim of the study was to study motor adaptation in a naturalistic table tennis paradigm and to investigate the influence of temporal order of perturbations (serial vs. random practice). We hypothesized that (a) typical motor adaptation effects can be observed. Furthermore, we hypothesized that (b) temporal order of perturbations (two distinct perturbations in alternating versus random order) influences (1) early adaptation, (2) late adaptation, (3) after-effects and (4) washout.

## Material and methods

### Participants

A total number of 30 right-handed healthy volunteers (average age: 22.93 ± 0.57 years; range 19–34 years; 14 women) were included in the present study. The study procedure was approved by the local ethics committee of the University of Leipzig (309/17-ek). All participants provided written informed consent and all procedures were conducted in accordance with the Declaration of Helsinki. None of the volunteers reported any previous neurological, psychiatric, cardiovascular, or musculoskeletal disease or took centrally acting drugs during the time of the experiment. Participants were randomly divided in two groups to differentiate motor adaptation effects comparing serial vs. random perturbation conditions. To ensure that both groups did not significantly differ in terms of potential confounders, (i) hours of sports per week, and (ii) hours of fine motor training per week were assessed (see Table 1). According to the Edinburgh Handedness Questionnaire^61^ all volunteers were right-handed (mean handedness score of 70.59 ± 3.30; cut-off score ≥ 50 indicated right-handedness; < 50 to >  − 50 indicate ambidextrous handedness; ≤  − 50 indicated left-handedness, Dragovic^62^). All participants were novice table tennis players who have never participated in regular table tennis training before. Nevertheless, participants had to be able to perform the required movement skill (backhand stroke) sufficiently well under predefined, simple conditions (without perturbations). For this reason, we used a pretest with a predefined target accuracy to ensure that participants met this prerequisite and successfully executed table tennis stroke movement (backhand stroke) under such conditions (see next section). A standardized questionnaire was used to assess (a) hours of sports per week and (b) hours of fine motor training per week (e.g. playing a musical instrument, knitting, handicrafts, playing video games). To control for possible psychological confounders, all participants assessed their attention (1 (very distracted)–10 (very attentive)), fatigue (1 (sleeping) – 10 (very energetic)), and discomfort (1 (no discomfort)–10 (strong discomfort)) on a visual analog scale (VAS) both before and after the entire experiment.

**Table 1:** Group demographics.

Group	Age (years)	Gender (female/male)	LQ (score)	Sports/week (hours)	Fine-motor training/week (hours)
Serial *n* = 15	22.07 ± 0.54	7/8	78.16 ± 4.31	5.63 ± 1.03	1.63 ± 0.46
Random *n* = 15	23.80 ± 0.97	7/8	76.46 ± 4.09	5.90 ± 0.72	3.97 ± 1.40

*LQ* laterality quotient as assessed with the Edinburgh handedness scale [range: − 100 (full left-handed) to + 100 (full right-handed)]. Hours of sports per week and hours of fine motor training per week (e.g. playing a musical instrument, knitting, doing handcrafts, playing video games with a keypad or joystick) were assessed with a questionnaire. All values are depicted as mean standard error (SE) of the mean. Statistical analysis revealed no differences in age, gender, LQ, sports/week, or fine-motor training/week between groups.

### Experimental procedure

**Figure 1:**
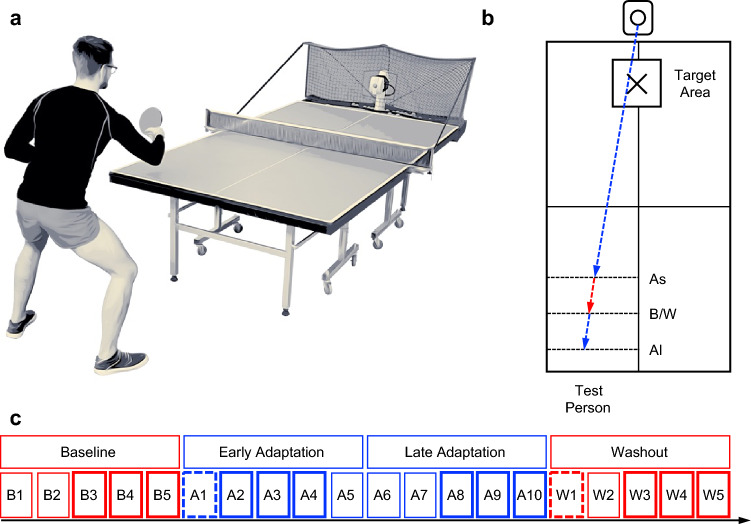
Study design and experimental setup. (**a**) Positioning of app-controlled ball robot at table tennis plate- Participant in backhand stroke (BH) position. (**b**) Positioning of topspin balls played by table tennis robot during baseline & washout (B/W: red dotted arrow). Backspin balls during adaptation (As/Al: blue dotted arrows) illustrated on table tennis plate with target cross & target area (pre-test accuracy threshold). (**c**) Experimental procedure: Twenty Blocks with 18 BH each (36 s) separated by 35–40 s jittered intertrial intervals. During five baseline blocks participants performed BH against easy-to-play topspin balls. During ten adaptation blocks participants performed BH against challenging backspin balls, representing disturbances in terms of motor adaptation. In the last five blocks (washout) BH must be performed against simple top spin ball, again. For statistical analysis of adaptation-related performance changes, mean distances of blocks B3 to B5 (baseline), A2 to A4 (early adaptation), A8 to A10 (late adaptation) and W3 to W5 (washout) were averaged (bold boxes). Mean distances of A1 represent initial error at perturbation onset and W1 represents after-effect after perturbation offset (bold dotted boxes).

Participants performed backhand strokes (BH) cross-court against balls played by an app-controlled table tennis robot (Donic Newgy Robo-Pong 3050XL, Germany) in a standardized manner (see Fig. 1a). For several reasons, we found that BH were more preferable than forehand strokes. In contrast to forehand stroke movement, table tennis racket is held in front of the body rather than next to it and stroke movement can be carried out with better control. Less degrees of freedom are involved in BH, which allows for a more stable posture. Since fewer movement artifacts are produced, this relatively stable position and reduced head movements are especially beneficial for future research considering the concurrent usage of neuroscientific research techniques like EEG and fNIRS.

Standardization includes spin type (topspin/backspin), spin strength, ball speed, height & placement of balls, number of balls (18) and wait time (2.00 s). During baseline phase (five blocks), BH were performed against easy-to-play topspin balls that hit the table tennis plate centered on backhand side at 90 cm from the net (see Fig. 1b). During adaptation, BH were performed against challenging backspin balls, which represent disturbances in terms of motor adaptation (ten blocks). In order to extend motor adaptation time, BH were performed against two distinct backspin balls of different lengths – a standardized ball that hit the plate at 55 cm from the net (As) and a standardized ball that hit the plate at 120 cm from the net (Al, see Fig. 1b). Due to the shorter time interval to react, the latter is more difficult to return. We hypothesized that temporal order of two differently placed backspin balls would affect target accuracy. Following literature on “contextual interference effect” in motor learning, we assumed that during practice context interference would be lower in serial order than in random order^43,44^. Aa a result, because of lower context interference, performance in acquisition phase should be higher. Accordingly, one group had to adapt to aforementioned backspin balls in alternating (“serial perturbation”, As-Al-As-Al) and the other group in randomized order (“random perturbation”).

During washout phase BH were performed against simple top spin balls, again (five blocks). BH strokes were executed according to a block design for 20 × 36 s (jittered intertrial interval 35–40 s, see Fig. 1c), whereby participants were instructed to perform strokes as accurately as possible. The target was marked with a target cross. In addition, a square target area was marked with side lengths of 40 cm (see Fig. 1b), which determines pre-defined target accuracy as part of a pre-test 24 h prior to main test (see previous section). For this purpose, participants had 5 blocks to place at least 9 of 18 balls per block in target area. Onsets of blocks were presented as auditory stimuli via Psychopy^63^. Motor adaptation was determined by means of target accuracy. For this purpose, 2D cinematography was used to determine the placement of participants’ balls. Subsequently, distances to target were calculated and adaptation-related changes in target accuracy were evaluated.

### Cinematographic analysis

Target accuracy was recorded with a high-speed video camera (GoPro Hero 9, San Mateo, US, 1920 × 1080 pixel, 200 frames per second, bird's eye view, about 70° shooting angle) placed on a tripod and evaluated offline. To improve illumination of the table tennis table and thus quality of video recordings, a spotlight was set up next to the camera. The white side lines along the 1.525 m and 2.74 m edge on the table were used to calibrate video recordings. Plane calibration (two-dimensional projective mapping) and measurements of ball placement on the table tennis table were done in Kinovea (v0.9.5), a free video annotation tool (GPL-2.0 license) designed for motion analysis that can be used to measure kinematic parameters. Furthermore, we performed an error detection routine for missed balls. We marked next to which quadrant of the table tennis table the balls were played (balls played too short on forehand (FH) or backhand (BH) side, ball played too long on FH or BH).

### Data analyses

In order to quantify dispersion of the balls played by table tennis robot, we recorded 100 balls (see Kinematics) in a preliminary study for each standardized ball type (baseline 90 cm, adaptation 55 & 120 cm). In the main study, Pythagorean Theorem was used to calculate distances between manually digitized hit points of participants’ balls and target point (center of target cross). Mean distance was obtained as a measure of target accuracy for each participant and each block. In addition, to represent the direction of deviations, centroids – arithmetic mean positions of all data points – were calculated for each subject and each block. Missed balls (that do not hit the table) are partially not visible in our recorded video images and thus cannot be captured cinematographically. For these balls, we had to set a fixed error for distance to target cross (150 cm), which is greater than the maximum deviation that occurs for a regular hit (127 cm). Furthermore, distribution of missed balls was modeled. We assume that a linear model with standard-normally distributed residuals describes the hit performances for each coordinate direction. The two parameters of the linear model and standard deviations were calculated for each coordinate direction. Normally distributed random numbers (0 ± SD) were added to the result of the linear equation. First values located in the corresponding quadrants were used (see Cinematographic Analysis/Error detection routine). This process was repeated up to the observed number of errors.

Data analysis and statistical analyses were performed using MATLAB (Version R2023a, MathWorks, Natick, MA, United States of America), RStudio (Version 2023.9.0.463, RStudio Team 2023) and JASP (Version 0.17, JASP Team 2021). For statistical analysis of adaptation-related performance changes, mean distances of blocks B3 to B5 (see Fig. 1c, baseline), A2 to A4 (early adaptation), A8 to A10 (late adaptation) and W3 to W5 (washout) were averaged. Mean distances of A1 represents initial error at perturbation onset and mean distances of W1 represents after-effect. Density plots are used to represent direction of deviations in target accuracy during baseline, initial error, adaptation, after-effects and washout. Density plots were created using bivariate kernel density estimation with 30 grid points in x/y. For perturbation groups (serial vs random), target accuracy during baseline, initial error, early and late adaptation, after-effects and washout was analyzed using a 6 (time) × 2 (group) mixed ANOVA with post-hoc tests (i.e., T-tests). If necessary, data were corrected for sphericity using Greenhouse–Geisser correction. Holm adjustment was used to counteract the problem of multiple comparisons. Threshold for statistical significance was set to α = 0.05.

## Results

### Behavioral data

**Figure 2:**
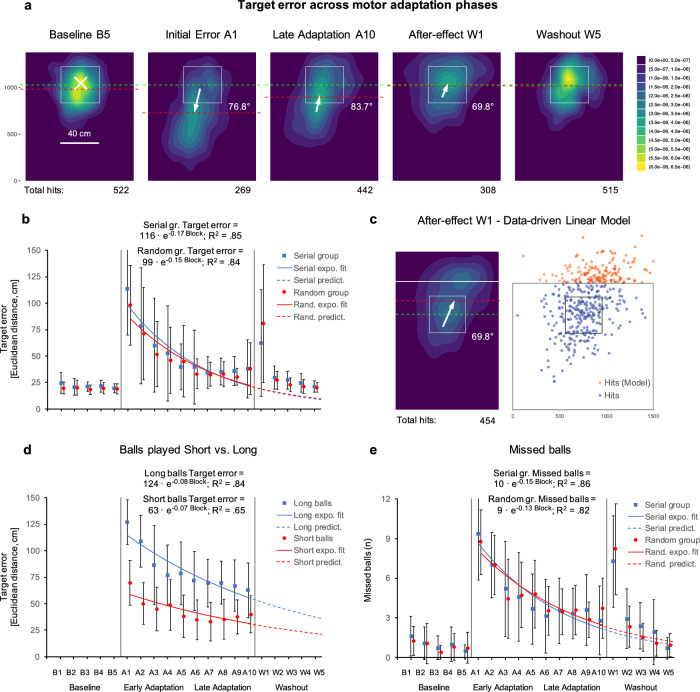
Motor adaptation assessed with Table Tennis Paradigm. (**a**) Density plots illustrate target error dispersions during Baseline (B5), Initial Error at Perturbation onset (A1), Late Adaptation (A10), After-effect (W1) and Washout (W5) as well as directional changes in target error dispersions from B5 to A1, from A1 to A10, and from A10 to W1 (white arrows & annotations). Colorbar values represent hit density (higher value represent a higher number of hits). Total hits: Number of balls hitting the table across all participants (out of 540 trials: 30 participants × 18 trials). Red dotted line represents average deviation in y-direction. Green dotted line represents target. (**b**,**d**) Dot plots and line plots (Exponential Curve Fit: y = a · e^b · x^ ; y = Target error, x = Block) showing adaptation-dependent performance changes in terms of spatial deviation from target (Euclidean distance) (**b**) for random and serial group, (**d**) for short and long balls. (**c**) After-effect (W1) Data-driven Linear Model. (**e**) Dot plots and line plots (Exponential Curve Fit: y = a · e^b · x^; y = Misses balls, x = Block) showing adaptation-dependent performance changes in terms of Missed balls (n) for random and serial group.

In terms of target accuracy, there was no interaction between time (baseline vs. initial error at perturbation onset vs. early vs. late adaptation vs. after-effects vs. washout) and perturbation groups (serial vs. random, *F*(2.57, 71.87) = 1.19, *p* = 0.317, *η*_*p*_^2^ = 0.04). We also identified no differences between perturbation groups (*F*(1, 28) = 0.11, *p* = 0.745, *η*_*p*_^2^ < 0.01). Thus, temporal order of perturbations did not influence motor adaptation. In contrast, we identified adaptation-dependent differences in target accuracy comparing baseline, initial error at perturbation onset, early and late adaptation, after-effects and washout across groups (*F*(2.57, 71.87) = 41.44, *p* < 0.001, *η*_*p*_^2^ = 0.60). Post hoc tests revealed an increase in target errors at perturbation onset (baseline (B3-B5)—initial error (A1), see Fig. 2b, *M*_*diff*_ = − 85.86, *t*(29) = − 11.80, *p*_*holm*_ < 0.001, *d* = − 2.79), significant early adaptation (initial error (A1)—early adaptation (A2-A4), *M*_*diff*_ = 45.69, *t*(29) = 6.28, *p*_*holm*_ < 0.001, *d* = 1.49) and late adaptation (early adaptation (A2-A4)—late adaptation (A8-A10), *M*_*diff*_ = 24.98, *t*(29) = 3.43, *p*_*holm*_ = 0.004, *d* = 0.81). We observed large target errors when perturbations ends (late adaptation (A8-A10)—after-effect (W1), *M*_*diff*_ = − 36.41, *t*(29) = − 5.00, *p*_*holm*_ < 0.001, *d* = − 1.18) and subsequently significant washout (after-effects (W1) – washout (W3-W5), *M*_*diff*_ = 48.45, *t*(29) = 6.66, *p*_*holm*_ < 0.001, *d* = 1.58). Furthermore, we didn’t find greater washout compared to early adaptation (*t*(29) = − 0.24, *p* = 0.815, *d* = − 0.04). In addition, we evaluated adaptation-related performance changes for short and long balls separately (see Fig. 2d & Supplemental Fig. 1). Compared to short balls, long balls initial error (A1) is higher (*t*(29) = 13.20; p < 0.001, d = 2.41), but adaptation-related changes are very similar (*t*(8) = -0.346; p = 0.738, d = − 0.12). Analysis of missed balls showed comparable results. There was no interaction between time and group (*F*(3.03, 84.88) = 0.60, *p* = 0.619, *η*_*p*_^*2*^ = 0.02), no differences between groups (*F*(1, 28) = 0.01, *p* = 0.917, *η*_*p*_^2^ < 0.01), but adaptation-dependent differences in missed balls comparing baseline (B3-5: 0.6 ± 0.2), perturbation onset (A1: 8.2 ± 0.7), early adaptation (A2-4: 5.0 ± 0.7), late adaptation (A8-10: 3.0 ± 0.5), after-effects (W1: 7.0 ± 0.7) and washout (W3-5: 1.3 ± 0.3) across groups (*F*(3.03, 84.88) = 88.34, *p* < 0.001, *η*_*p*_^2^ = 0.76, see Fig. 2e & Supplemental Fig. 2).

Density plots (see Fig. 2a) illustrate target accuracy dispersions during baseline, initial error (at perturbation onset), late adaptation, after-effects and washout as well as adaptation-induced directional changes in target accuracy dispersions. During perturbation onset majority of balls were played too short resulting in large target errors (Centroids *C*: *C*_B3-B5_–*C*_A1_: 0.25 m, 76.8°, see Fig. 2a). In contrast, when perturbation disappears, majority of balls were played too long resulting in target errors in opposite direction (*C*_A8-A10_–*C*_W1_: 0.14 m, 69.8°, see Fig. 2a).

Furthermore, during AE/W1 the majority of missed balls are played too long (n = 162 too long vs. n = 5 too short; balls played too long FH: n = 111, BH: n = 51). Data-driven linear model (see Fig. 2c) shows that density plot AE/W1 (block 16, Fig. 2a) underestimates the after-effect.

### Psychological and physiological confounders

Statistical analysis of questionnaires revealed an increase for participants’ attention (pre: 7.0 ± 1.0 [median ± MAD], post: 7.5 ± 1.5, z = − 2.13, p = 0.031) and fatigue level (pre: 7.0 ± 2.0, post: 7.5 ± 1.0, z =  − 2.66, p = 0.007). Regarding discomfort, there was no pre-post difference (pre: 1.0 ± 0.0, post: 1.0 ± 0.0, z = 1.68, p = 0.105). There were no statistically significant differences between perturbation groups (serial vs random) concerning pretest (attention: p = 0.471, U = 130.0, fatigue: p = 0.240, U = 141.0, discomfort: p = 0.872, U = 116.5), posttest (attention: p = 0.269, U = 139.0, fatigue: p = 0.611, U = 125.0, discomfort: p = 0.735, U = 120.0), or pre–posttest differences (attention: p = 0.816, U = 106.5, fatigue: p = 0.932, U = 115.0, discomfort: p = 0.458, U = 99.5).

## Discussion

The goal of motor adaptation research is to better understand and enhance the impressive, mostly implicit human adaptability to changing environmental conditions. Until now behavioral and neurobehavioral studies only investigated artificial and highly controlled motor tasks. Currently, there is only sparse knowledge about the occurrence of motor adaptations in more natural motor tasks performed under externally valid conditions. In this context, our study aimed to evaluate motor adaptation during table tennis and investigated the impact of the temporal order of perturbations which were presented either in serial or random order. Based on numerous studies investigating motor adaptation^15,16,21,64^, we hypothesized that typical motor adaptation effects can be observed during table tennis. Furthermore, we hypothesized that temporal order of perturbations (two distinct perturbations in alternating versus random order) influences early adaptation, late adaptation, after-effects and washout^43,44^.

Consistent with our first hypothesis, we observed an increase in target errors with perturbation onset that decreased during motor adaptation. Furthermore, we observed an increase in target errors with perturbation offset (after-effect) that decrease subsequently during washout phase. Furthermore, we analyzed adaptation-induced directional changes in target accuracy dispersions. We observed typical after-effects pointing in opposite direction compared to initial error at perturbation onset. This discovery might be associated with the presence of an adapted internal model that initially persists when perturbation ends^16^. Contrary to our second hypothesis, temporal order of perturbations did not affect motor adaptation. In the following, we will discuss our results in more detail.

First, we shed light on the observed adaptation-induced changes in target accuracy and directional changes in target accuracy dispersions. Generally, participants had to adapt the angle between their racket and the horizontal plane in order to successfully perform the motor task (backhand strokes). During baseline and washout phase, participants had to return standardized topspin balls played by the ball robot. For a successful return, the table tennis racket had to be "closed”. “Closed” in this case means, that the racket is tilted forward. Thus, the upper edge of racket pointed in stroke direction. At the beginning of adaptation, backspin balls hitting a still “closed” racket caused them to bounce downward and thus they were played too short. In the course of adaptation racket had to be "opened", so that balls get our intended distance. “Opened” means that the racket was tilted backwards. Thus, the lower edge of racket pointed in stroke direction. At the beginning of washout, topspin balls hitting a still "opened" racket caused them to bounce upwards and thus they were played too long. In the course of washout, racket had to be "closed" again, so that balls get necessary distance, again. The observed gradual increases in target accuracy suggest that novices implicitly adjusted racket angle during adaptation process. It can be assumed that advanced players, who already gained explicit knowledge about required racket angle changes, adapt more quickly to this procedure since they are able to anticipate ball spin and continuously adjust their racket angle. Future studies could examine how target accuracy differs among novices who received explicit knowledge and novices who did not receive explicit knowledge (as in our study). Furthermore, our observations can also be attributed to the change from constant to variable conditions. To reduce results to a single dimension, we evaluated adaptation-related performance changes for short and long balls separately. In summary, typical adaptation-related changes are present for both, short and long balls. Compared to short balls, long balls allow only a short movement preparation. Initial error is higher, but adaptation-related changes are very similar. In summary, two-dimensional adaptations are present in our study—adaptations to spin and adaptations to the change from constant to variable conditions. However, we believe that sport-specific perturbations always require multidimensional adaptations and our study represents such adaptations at least partially. Moreover, we show that systematic directional changes during the adaptational process are caused mainly by the adaptations to spin.

Regarding our second hypothesis, we observed that the temporal order of perturbations did not affect the amount of motor adaptation. Initially, we assumed that context interference would be lower for serial order compared to random order^43,44^. Accordingly, performance improvements during motor adaptation should be superior in serial group (lower context interference) compared to random group (higher context interference). In fact, a recent meta-analysis^65^ reports that there is incongruency in the current literature regarding knowledge about the amount of contextual interference that results from practicing in serial order. The meta-analysis thus confirms authors who suggest that serial practice (as well as randomized practice) involves high contextual interference and leads to high contextual interference effects^66–68^. The findings of our study confirm these observations for motor adaptations. Furthermore, the random order condition may have provided less interference between trials due to the lower number of task switches. Specifically, there were eight task switches between short and long balls. In contrast, due to the constant changes, there were 15 task switches in the serial order group. Future studies should use a standardized number of e.g. eight task switches in the serial order and random order group. Furthermore, future studies on temporal order of perturbations should compare serial or randomized practice with blocked practice. Context interference should be lower for blocked practice^69,70^.

The current study faces some limitations. Examination of target accuracy by cinematography is very time-consuming in this study. Each hit point in our captured videos needed to be located and manually digitalized. This analysis-step should be automated in future investigations. Additionally, it has to be considered that balls can bounce back from ball robot and catch-net. Furthermore, missed balls (that do not hit the table) were partially not visible in our recorded video images and could therefore not be captured cinematographically. For these balls, a fixed error for distance to target was set (see method section). Changing this fixed error induces changes in the level of observed target accuracy. However, it is important to note that adaptation-related temporal changes in radial error, which were our primary interest, were not affected (see Supplemental Fig. 3). Nevertheless, direction-related analysis of target accuracy could not be performed for these missed balls. As a result, directional analysis (density plots, see Fig. 2a), which could only include balls that hit the plate, underestimates average distance to target. For this reason, distribution of missed balls was modeled. Here, modeling shows that density plot underestimates the after-effect. Therefore, we are confident, that our paradigm reflects typical motor adaptation in a sport-specific context. In order to minimize the number of missed balls, we conducted a pre-test 24 h before our main study. This pre-test should secure that participants are able to perform required movement skill sufficiently well during baseline (without perturbations). Furthermore, a kinematic observation, especially of the adaptation-related changes in hand movement (inclination of the table tennis racket), would give additional information about the learning process and should therefore be incorporated in future studies.

Future studies should shed more light on the effect of the temporal order of perturbations in more naturalistic settings. It is reasonable to assume, that a blocked order would affect motor adaptation more prominently as compared to serial or random order^69,70^. Furthermore, the effect of motor expertise should also be investigated more thoroughly. Table tennis athletes, for example, should show significantly lower target errors and should adapt to perturbations much faster as compared to novices. How this pattern is affected by the temporal order of perturbations is still debatable. Additionally, it would be of interest to quantify neurophysiological changes during motor adaptations in sport-specific settings. A better understanding about the underlying mechanisms could be used to optimize training regimes and to reduce the motor adaptation process.

### Supplementary Information

Supplementary Figures.

## Data Availability

All data that support the findings of this study are available from the corresponding author, D.C. if a formal data sharing agreement exists. Besides, all software used in the present study is open-source and as such publicly available.
