# Supplementary material for: Quantifying motor adaptation in a sport-specific table tennis setting
Source: Sci Rep. 2024 Jan 5;14:601. doi: 10.1038/s41598-023-50927-2 (PMC10770152; doi:10.1038/s41598-023-50927-2)
Supplement: Supplementary file 1 — Supplementary Figures. [file 41598_2023_50927_MOESM1_ESM.docx]

Quantifying motor adaptation in a sport-specific table tennis setting (Daniel Carius et al.) Supplemental Figure 1

| **** |
| --- |
| **Supplemental Figure 1:** Adaptation-dependent performance changes (spatial deviation from target) **for short and long balls**. Exponential Curve Fit (upper panel, y = a · e^b · x^ ; y = Target error, x = Block). Log transformed (ln) results (lower panel). |

Quantifying motor adaptation in a sport-specific table tennis setting (Daniel Carius et al.) Supplemental Figure 2

| **** | |
| --- | --- |
| **Supplemental Figure 2:** Adaptation-dependent performance changes in missed balls for **serial and random group**. Exponential Curve Fit (upper panel, y = a · e^b · x^ ; y = Missed balls, x = Block). Log transformed (ln) results (lower panel). |  |

Quantifying motor adaptation in a sport-specific table tennis setting (Daniel Carius et al.) Supplemental Figure 3

|  |
| --- |
| **Supplemental Figure 3:** Adaptation-dependent performance changes (spatial deviation from target). Comparative plot showing a change in fixed **error from 150 to 200 cm.** Exponential Curve Fit (upper panel, y = a · e^b · x^ ; y = Target error, x = Block). Log transformed (ln) results (lower panel). |
